# USE OF GASTROSCOPE FOR EVALUATION AND TREATMENT OF LESIONS IN THE PROXIMAL JEJUNUM

**DOI:** 10.1590/0102-672020180001e1446

**Published:** 2019-08-26

**Authors:** Vishal SHARMA, Sandeep LAMORIA, Shashi DHAWAN, Arka DE, Brinder Mohan S LAMBA

**Affiliations:** 1 Department of Gastroenterology, Dr RML & PGIMER; 2 Department of Pathology, Sir Ganga Ram Hospital, Delhi, India

**Keywords:** Intestine, small, Anti-inflammatory agents, non-steroidal, Giardia, Balloon enteroscopy, Intestino delgado, Anti-inflamatórios não esteroides, Giardia, Enteroscopia de balão

## INTRODUCTION

Small bowel is a difficult area to visualize with endoscopy. While ileo-colonoscopy can help visualize the terminal few centimeters of the ileum, esophagogastroduodenoscopy is usually utilized to view the gastrointestinal tract till the proximal duodenum. The visualization of distal duodenum, jejunum and ileum requires advanced techniques. While capsule endoscopy can provide the visualization of the entire small bowel, it is costly and cannot be used to obtain tissue for histology or for therapeutic purpose. Enteroscopic techniques like the push enteroscopy, spiral enteroscopy and single or double balloon enteroscopy are used to diagnose and treat small bowel lesions^1^. However, these are costly and their availability is scarce as is the expertise in their use. The accessories for their use are different and add to the cost of therapy. Previously the use of pediatric colonoscopes has been reported for push enteroscopy^2^. However, the more readily available adult colonoscope may not be helpful because of the larger diameter. We hypothesized that the gastroscope may be used to access the proximal jejunal lesions. 

## METHOD

The patients who were suspected to have lesions in the proximal jejunum underwent an attempt at enteroscopy with use of gastroscope (Olympus GIF-180). The usual endoscopy was done to reach the second part of duodenum. During this excessive insufflation was avoided especially in the stomach and the gastroscope was rapidly advanced across the pylorus to avoid distension of the stomach. Once the gastroscope was in the second part of duodenum, it was reduced by withdrawing it. After this with use of suction to ensure that the duodenal lumen does not overdistend, the gastroscope was advanced as in push enteroscopy. If looping occurred, the assistant applied pressure along the greater curvature of the stomach and the gastroscope was advanced into the jejunum till the available gastroscope length or till the lesion was reached. 

## REPORT OF CASES

### Case 1

**FIGURE 1:**
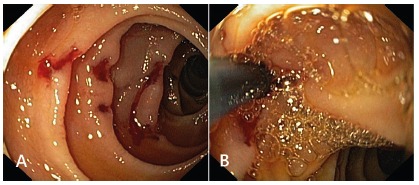
A) Bleeding jejunal ulcers; B) cessation of bleeding with injection therapy

A 67 year old lady a known diabetic for 10 years and now having symptomatic bilateral knee osteoarthritis presented with history of melena, progressive exertional dyspnea and generalized weakness for one month. She had used multiple non-steroids anti-inflammatory drugs (NSAIDs) including diclofenac and aceclofenac for her joint pains. Her hemoglobin at presentation was 5.7 gram/dl and she was stabilized with transfusion of packed red cells. Her initial upper endoscopy and colonoscopy were normal. In view of ongoing melena we used gastroscope to visualize the jejunum and multiple actively bleeding ulcers were noted in the proximal jejunum. Injection therapy with adrenaline was done to achieve hemostasis (Figure 1A and 1B). The patient improved and was discharged with advice to avoid NSAIDs. At a follow-up visit one month later the lady had improved and her hemoglobin was 10.3 gram/dl.

### Case 2 

**FIGURE 2:**
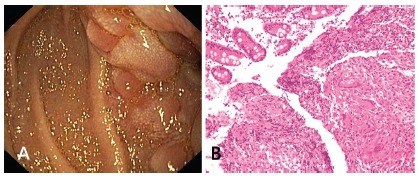
A) Abnormal and thickened jejunal folds; B) - Histology showing well formed non necrotizing granuloma in the lamina propria (H&E 200X)

A 48 year old lady was symptomatic for two months with recurrent episodes of abdominal pain associated with vomiting and loss of weight and appetite. She also reported evening rise of temperature and night sweats. Her hemogram was suggestive of anemia (Hb: 8.9 gram/dl, TLC: 8900, platelet: 3.2 lakh/mm^3^. Mantoux test was positive (>10 mm). Abdominal computed tomography revealed mural thickening of jejunal and ileal loops. Ileo-colonoscopy was normal. On upper endoscopy a narrowed area with thickened fold of the jejunum (Figure 2A) was noted and multiple biopsies were obtained. Histology revealed presence of non-necrotising granulomas (Figure 2B). The patient was initiated on four drug anti-tubercular therapy and improved with it. After six weeks the patient had gained 3 kg of weight and had improvement in abdominal pain and fever. 

### Case 3 

**FIGURE 3:**
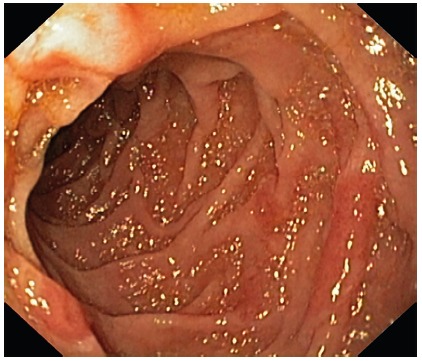
Focally denuded mucosa in jejunum

A 27 year old manual laborer was evaluated for abdominal pain, loose stools and weight loss. The patients had been symptomatic for six months. The work-up including HIV serology and abdominal ultrasound and chest X-ray were normal. His hemoglobin was 9.8 gram/dl and leucocyte and platelet count was normal as were his renal and liver function tests. Abdominal computed tomography had revealed mild mural thickening of jejunal loops. Endoscopy was normal and therefore we did jejunal examination using gastroscope which revealed focally denuded villi (Figure 3) from which multiple biopsies were taken. The histology revealed multiple trophozoites Giardiasis and the patient improved with a 7-day course of oral metronidazole. 

## DISCUSSION

We hereby have described the use of gastroscope to access three patients having lesions in the proximal jejunum. The method may provide an opportunity for endoscopists with limited availability of enteroscopy to access, sample and treat lesions in the proximal jejunum. However, this comes with a caveat that the gastroscope cannot be used for distal jejunal or ileal lesions for which other methods like push, spiral or balloon enteroscopes have to be used. 
